# Soil moisture variations affect short-term plant-microbial competition for ammonium, glycine, and glutamate

**DOI:** 10.1002/ece3.1004

**Published:** 2014-03-06

**Authors:** Katarina F Månsson, Magnus O Olsson, Ursula Falkengren-Grerup, Göran Bengtsson

**Affiliations:** Department of Ecology, Lund UniversityEcology Building, SE-223 62, Lund, Sweden

**Keywords:** ^13^C-^15^N-amino acids, ^15^N, deciduous forest, *Festuca gigantea*, root, soil drying–rewetting

## Abstract

We tested whether the presence of plant roots would impair the uptake of ammonium (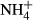), glycine, and glutamate by microorganisms in a deciduous forest soil exposed to constant or variable moisture in a short-term (24-h) experiment. The uptake of ^15^NH_4_ and dual labeled amino acids by the grass *Festuca gigantea* L. and soil microorganisms was determined in planted and unplanted soils maintained at 60% WHC (water holding capacity) or subject to drying and rewetting. The experiment used a design by which competition was tested in soils that were primed by plant roots to the same extent in the planted and unplanted treatments. *Festuca gigantea* had no effect on microbial N uptake in the constant moist soil, but its presence doubled the microbial 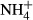 uptake in the dried and rewetted soil compared with the constant moist. The drying and rewetting reduced by half or more the 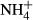 uptake by *F. gigantea*, despite more than 60% increase in the soil concentration of 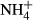. At the same time, the amino acid and 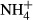-N became equally valued in the plant uptake, suggesting that plants used amino acids to compensate for the lower 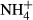 acquisition. Our results demonstrate the flexibility in plant-microbial use of different N sources in response to soil moisture fluctuations and emphasize the importance of including transient soil conditions in experiments on resource competition between plants and soil microorganisms. Competition between plants and microorganisms for N is demonstrated by a combination of removal of one of the potential competitors, the plant, and subsequent observations of the uptake of N in the organisms in soils that differ only in the physical presence and absence of the plant during a short assay. Those conditions are necessary to unequivocally test for competition.

## Introduction

Observations of plant uptake of organic nitrogen (N), particularly amino acid N, in soils with large amounts of organic N but insufficient net mineralization rates to satisfy the annual N demand (e.g., Schimel and Chapin 1996; Näsholm et al. 1998) have refueled the debate on the competitive interaction between plants and soil microorganisms (Kaye and Hart 1997). As plants from ecosystems with relatively high net N mineralization rates also take up amino acids (Näsholm et al. 2009), it is possible that organic N may be another shared and mutually limiting N source over which plant–microbial competition may occur, especially during periods when inorganic N is scarce.

Competition for N may come out differently depending on the access to various N sources, their concentration in the soil (Bardgett et al. 2003), and other soil conditions, such as spatial distribution of roots and microorganisms (Wang and Bakken 1997; Hodge et al. 2000; Xu et al. 2011), decomposability of the soil C (Månsson et al. 2009), and soil moisture (Schimel et al. 1989; Lipson and Monson 1998). However, little is known about the conditions favoring the uptake of mineral N versus amino acid N in plants and microorganisms. The ratio of glycine-to-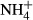 uptake varies from 0.2 to 7.7 in microorganisms and from 0.6 to 2.1 in plants, in arctic systems (Schimel and Chapin 1996; Henry and Jefferies 2003) and temperate grasslands (Bardgett et al. 2003). In the latter system, the microbial uptake was dominated by glycine during plant growth in May but shifted to 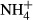 in September, possibly in response to an increased pool of easily decomposable C as plant senescence started. This preference for 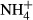 to glycine in microorganisms was also evident in their response to glucose addition to soil, which increased respiration, at the same time as grasses in the same soil reduced their uptake of glycine more than the uptake of 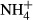 (Dunn et al. 2006). When soil moisture conditions are constant, microorganisms show no uptake discrimination between 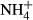 and amino acids or prefer simple amino acids to 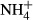, whereas most grass species and shrubs seem to prefer 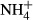 and glycine to more complex amino acids, although species differences exist (Weigelt et al. 2005; Harrison et al. 2007; Sørensen et al. 2008).

Short drying and rewetting cycles occur frequently during a growth period in temperate forests (Ladekarl 1998; Subke et al. 2003). When the soil is drying, bacteria accumulate osmotic active internal solutes, such as free amino acids and their derivates, to maintain the internal water potential in balance with the surrounding environment (Csonka and Hanson 1991). Following rewetting, concentrations of amino acids and mineralized N increase in the soil due to cell lysis (Marumoto et al. 1982; Pulleman and Tietema 1999) and microbial decomposition of soil organic matter (Van Gestel et al. 1993; Appel 1998; Lipson and Monson 1998).

As the soil is rewetted, the efflux of easily decomposable C from roots (Neuman and Römheld 2001), microorganisms (Marumoto et al. 1982), and soil organic matter (Pulleman and Tietema 1999) will increase. This easily available C may fuel fast-growing microorganisms (Bottner 1985; Van Gestel et al. 1993) and increase rates of microbial N mineralization and net immobilization of 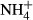 (Schimel et al. 1989; Pulleman and Tietema 1999; Bengtsson et al. 2003; Kaiser et al. 2011), to the detriment of N uptake in plants. Under constant and high soil moisture conditions, fast-growing microorganisms will be less active (Bottner 1985; Van Gestel et al. 1993) and possibly open a window of competition with plants for limited quantities of mineralized N.

Plants can also respond rapidly to rewetting of a dry soil and restore the 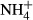 and NO_3_^−^ uptake within hours to a few days after moderate-to-severe drought (Brady et al. 1995; Cui and Caldwell 1997; Buljovcic and Engels 2001). Furthermore, organic N is potentially a more cost-effective N source than inorganic N (Schmidt and Stewart 1999), e.g., by also supplying carbon to a plant that has been energy limited during drought (Raab et al. 1996).

According to the resource competition model (Burkholder 1952), competition for the same and limiting resource is assumed to bear a cost to all competitors. Both plants and microorganisms qualify for part of the definition, as they can use the same sources of N. However, just like removal, introduction or both of one or more potential competitor is a necessary criterion to demonstrate competition (Schoener 1983). Solely relying on uptake data, as in most work on plant-microorganism competition for nitrogen sources so far, is not a proof for plant–microbial competition for N. But uptake data will, in combination with the removal of one or the other group of organisms, be useful in testing the second part of the condition for resource competition, namely the limitation of the availability of N by one potential competitor to the other. Excluding the microorganisms from a competition assay in soil without changing the soil conditions is not an alternative, so we made an effort to exclude the plant and yet provide the same soil conditions as if the plant had been present.

To address the issue of competition for different N forms between plants and microorganisms under varying moisture regimes, we developed two hypotheses to test in this project:

1. Microorganisms will take up less 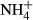 in the presence than in the absence of the plants in a constantly moist soil.
2. Immediately following rewetting of a dried soil, microorganisms increase their total uptake of N, especially 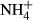, in response to the increased N pool in the soil, while plants turn to an increased uptake of energy providing N sources, that is, glycine and glutamate.

To test the hypotheses, plant-microbial competition for 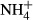, glycine and glutamate, which represent less and more complex amino acids, respectively, was studied in a 24 h assay, in which the ^15^N uptake in the organisms was measured in planted soils and unplanted reference soils that had been either constantly moist or dried and rewetted over a short period of time. Dual labeled amino acids (^13^C-^15^N-amino acids) were used to estimate the uptake of intact amino acids by the plants. The soil ATP content was analyzed to estimate the soil microbial biomass and activity.

## Material and Methods

### Soil

The soil was classified as dystric cambisol (FAO system; soil characteristics are given in Table 1) and was sampled at Torup, southern Sweden (55°33^′^N, 13°37^′^E). Oak (*Quercus robur* L., 80%) and beech (*Fagus sylvatica* L., 20%) were dominating the tree canopy, and the understory vegetation was sparse. Litter was removed within a 20 × 20 m square, and five soil samples, each covering a 0.5 × 0.5 m square, were randomly collected from the top 5 cm, sieved through a 4-mm mesh, and then pooled. The soil C:N ratio, pH (H_2_O), and water holding capacity (WHC) were determined. The soil was stored at 2°C until planting 2 weeks after sampling.

**Table 1:** The characteristics of the soil used in the experiments. The pH, soil total C and N (mg g^−1^ dw soil), soil C:N ratio, and field concentrations (*μ*mol/L) of glycine, glutamate, sum of all analyzed amino acids (Total a.a.), and 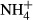 in the soil solution sampled with Rhizon tension lysimeters (means ± SE, *n *=* *5) when the samples for the experiment were collected. Total a.a. shown after decreasing concentrations: glutamine (43.8 *μ*mol/L), aspartic acid, glutamate, alanine, aspargine, serine, glycine, threonine, and arginine (2.4 *μ*mol/L).

pH (H_2_O)	Soil C	Soil N	Soil C:N	Concentrations of amino acids and 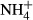			
				Glycine	Glutamate	Total a.a.	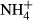
3.5	57	2.9	20	16.2 ± 2.3	25.9 ± 1.9	190.0 ± 11.4	189.9 ± 44.7

Values are mean ± SE (*n *=* *5).

### Plant material

We used *Festuca gigantea* L., a common grass in the type of oak–beech forest where the soil was sampled. *F. gigantea* grows relatively fast (RGR = 1.0–1.4 week^−1^; Grime et al. 1988) and is often found on soils with relatively high N mineralization and nitrification rates, as indicated on a 1–9 scale by its Ellenberg N value of 6 (Ellenberg et al. 1991) and FNIS value of 8 (calculated from data in Diekmann and Falkengren-Grerup 1998). FNIS values are taking both the total amount of mineral N and the amount of NO_3_^−^ into consideration, that is, when two soils have the same concentration of total mineral N, the soil with the highest NO_3_^−^ concentration will get the highest FNIS value. *F. gigantea* is most often found to be nonmycorrhizal (Harley and Harley 1987). The plants were grown from seeds in >99% pure silica sand supplied with a nutrient solution (including 150 *μ*mol/L NO_3_^−^ and 100 *μ*mol/L 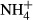) for one to 2 weeks after germination and then planted in the experimental soils. Seedlings included in the experiment had a shoot height of 7–10 cm and root length of 5–8 cm.

### Experimental design and ^15^N addition in the competition assay

Before planting, the field moist soil was wetted with deionized water to obtain 60% WHC 1 day before planting. Transparent plastic pots with a diameter of 27 mm and a height of 100 mm were filled with 84 g of soil (62 g dry weight soil). The pots were placed in a greenhouse at a temperature of 20°C day/16°C night and 16 h of daylight (with additional light of 160 *μ*mol m^−2^ s^−1^ during the day). Sprinklers maintained the relative air humidity at about 50%. The pots were kept at 60% of WHC by addition of deionized water.

**Figure 1:**
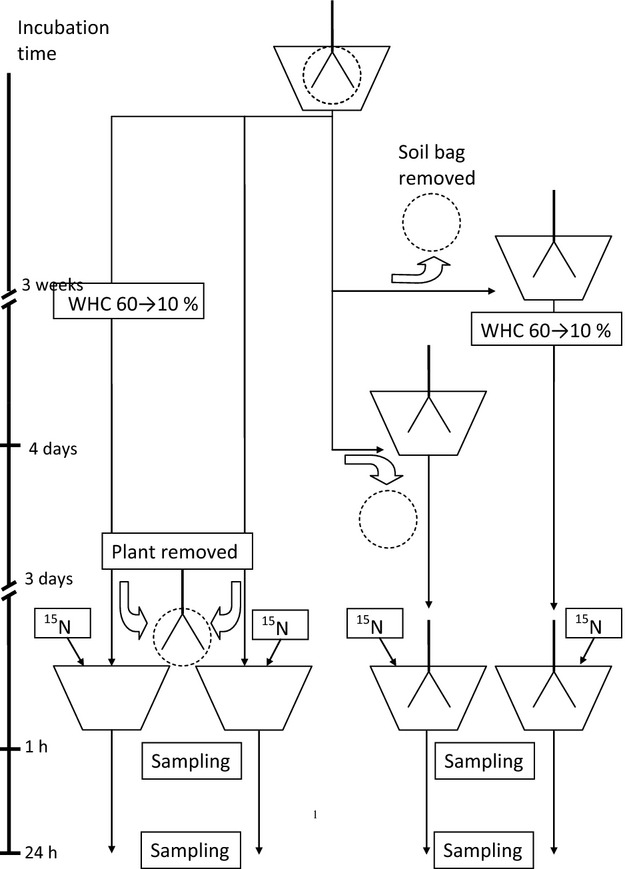
Schematic drawing of the experimental design of the plant–microbial competition assay. The plant with its roots enclosed in a soil bag was incubated for 3 weeks in pots filled with soil. Then, the soil bag was removed in one-fourth of the pots, the plant returned to the soil, and the water holding capacity (WHC) adjusted from 60% to 10 in 4 days. At that time, the soil bag was removed in another fourth of the pots, and the plant returned to the soil. The plants with removed soil bag were incubated for another 3 days, one-fourth at 10% WHC and the other fourth at the original 60%. The third-fourth of the pots were incubated for 3 weeks plus 4 days plus 3 days at the original 60% WHC, with the roots enclosed in a soil bag. The fourth-fourth of the pots was incubated for 3 weeks, then, WHC in the soil was adjusted from 60% to 10 for 4 days, and the plant with roots enclosed in a soil bag incubated for another 3 days at 10% WHC. The plant in the last two treatments was then removed from the pot and the soil in the soil bag returned to the bulk soil in the pot. The ^15^N labeled amino acids were added to the soil in all four treatments, samples were taken within 1 h for determination of the initial ^15^N values and after 24 h for the final values.

We developed a new competition assay method, in which also the soil that was used as unplanted in the assay was planted prior to the assay, to ensure that the effect of plant presence on competition would be measured with an equal biomass and activity of microorganisms and equal concentrations of dissolved C and N in the planted and unplanted soil. For this purpose, two seedlings of *F. gigantea* were grown in each pot for 3 weeks with the roots of each plant enclosed with soil in nylon bags (width 15 mm, height 50 mm, and mesh size 25 *μ*m) (Fig. 1). The bags separated the roots physically from the bulk soil but allowed, for example dissolved N and C and microorganisms to move between the bulk soil and the soil in the root bag. The root bags also enabled us to remove the plants from the soil without leaving root fragments.

After 3 weeks of incubation, the pots were divided into two main groups. One of them (40 pots) was prepared to house the plants during the ^15^N competition assay. This group was divided into two subgroups, with 20 pots in each. In one of them, the root bag with the two individuals of *F. gigantea* was removed from the bulk soil. The plants were carefully taken out of their bag. Both individuals of *F. gigantea* were replanted in the hole left after the bag, the soil from the bag was returned into the hole, and the soil surface carefully compacted to avoid preferential flow of the labeled solution to be added later. The pots with the replanted plants were kept in the greenhouse to adjust soil moisture to 10% of WHC, which was obtained after 4 days. The plants were then kept at 10% of WHC for another 3 days. One hour before ^15^N addition, WHC was returned to 60% by addition of deionized water.

In the other subgroup, the plants were kept in the greenhouse at a constant 60% of WHC, replanted 3 days before ^15^N addition, as described, and kept at 60% of WHC until ^15^N was added.

The other main group of pots, prepared to be unplanted during the assay, was also divided into two subgroups, each with 20 pots. In one of the subgroups, WHC of the soil was adjusted to 10% in 4 days and kept at 10% of WHC for another 3 days, as described above. The two individuals of *F. gigantea* were then removed, the roots gently separated from the soil in the root bag, the soil returned into the hole left after the bag, and the soil surface gently compacted. One hour before ^15^N addition, WHC was returned to 60% by addition of deionized water.

The 20 pots in the other subgroup prepared to be unplanted during the assay were kept in the greenhouse at a constant 60% of WHC until the day for ^15^N addition. Then, the plants were removed from the soil as described above, the soil returned to the hole left after the bag, the soil surface gently compacted, and the plants harvested for analysis, as described below.

Ten replicate pots for each treatment received deionized water spiked with 0.05 *μ*mol ^15^N per g dw soil as ^15^NH_4_Cl, sodium glutamate (H_2_^15^N^13^CO[^13^CH_2_]_2_[^13^COONa]_2_) or glycine (H_2_^15^N^13^CH_2_^13^COOH) (Cambridge Isotopic Laboratories, >98% ^15^N). Four 0.5-mL injections per pot were made with a 10-cm long syringe, which was gradually withdrawn from the bottom to the top of the pot to distribute the solution evenly.

Within 1 h after first injection, half of the number of treated pots was harvested as described below, and the initial (*t* = 0) ^15^N (atom%) in soil and plants determined. These data were used in calculating the gross uptake of N in organisms (see section Calculations) and for comparison of plant ^15^N with that in plants harvested from pots prepared to be unplanted. The remaining pots were left for 24 h in a climate chamber (20°C/15°C day-night temperature) with 16 h of light of 400 *μ*mol m^−2^ s^−1^ and a mean air humidity of 50%.

When harvested, the plants were gently removed from the soil and rinsed in a mixture of 1.0 mmol/L KCl and 0.5 mmol/L CaCl_2_ for 5 min to remove ^15^N from the root surface. The plants were then dried at 70°C for 24 h. The dried roots and shoots were separately weighed and ground into a fine powder using a ball mill (Retsch, Mixer Mill 200).

Samples of 10 g of gently mixed soil (soil bag and surrounding 6 mm wide casing of soil) from the upper 50 mm of a pot were taken for chloroform fumigation-extraction to release the ^14^N and ^15^N in the microbial biomass. The extraction followed the method of Brookes et al. (1985), except that 0.4 mol/L KCl was used instead of 0.5 mol/L K_2_SO_4_, to maximize the amount of N per g salt in the soil extract. Five mL of the 0.4 mol/L KCl soil extracts from fumigated and unfumigated samples was vacuum centrifuged without heat in a centrifuge (Savant AES 1000) to remove water, and the residue was ground using a mortar and pestle. The dried plant and soil samples were then analyzed with isotope ratio mass spectrometry to determine total ^15^N (atom%) and concentrations, as described below.

To estimate the potential assimilation of ^13^CO_2_ by plants during the 24 h incubation, control pots with *F. gigantea*, receiving only deionized water, were placed among the pots receiving ^13^C^15^N-amino acids. No detectable ^13^C enrichment was found in those plants (data not shown), suggesting that assimilation by plants of ^13^CO_2_, which may have been released from pots treated with labeled amino acids, was negligible.

### Analysis of ^15^N and ^13^C with mass spectrometry

The ground, dried plant material of roots and shoots and the homogenized salt from dried soil extracts were weighed in tin capsules (4 mg plant material and 80 mg salt) and analyzed for ^15^N and ^13^C with continuous flow isotope ratio mass spectrometry. The samples were oxidized in an ANCA-GSL elemental analyzer and passed to a 20-20 isotope ratio mass spectrometer (IRMS, PDZ Europa, UK). The amount of total N and C was quantified, after the subtraction of values from blank samples, using standard curves of 5, 50 and 100 *μ*g N as glycine. One reference sample (glycine) was analyzed after every fifth sample to compensate for drifting isotope ratios. The *δ*^15^N of the reference was calibrated against atmospheric N_2_ and the *δ*^13^C against PDB. The precision of the isotopic determinations was <0.2 ‰ for N and <0.1 ‰ for C.

The total amount of N in the soil extracts was too low (often <10 *μ*g N per sample) to be determined with sufficient precision by the mass spectrometer. Therefore, it was determined by a Shimadzu TOC-V_CPH_ analyzer (Kyoto, Japan) after the extracts had been diluted five times with deionized water to reduce the salt interference.

### Analysis of soil amino acids and
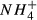


The soil water was sampled with Rhizon tension lysimeters (Rhizon SMS, Wageningen, the Netherlands). Recent studies suggest that samples from small tension lysimeters give a more representative description of the soluble N pool available for plant acquisition and the contribution of amino acids relative to 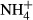 to the pool of plant available N than common extraction methods with water or salt solutions (Inselsbacher et al. 2011). The lysimeters were inserted before the rewetting, and samples were taken 1 h after rewetting the dry soil in both the dried–rewetted and the constantly moist treatment. The pots were treated in the same way as the pots in the competition assay but without addition of ^15^N and were sampled at the start of the assay. The lysimeter (diameter 2.5 mm, length 50 mm) was made of a porous polymer with a mean pore size of 0.1 *μ*m. The small pore size in combination with the sterile sampling tube minimized the microbial activity in the samples. The lysimeter was installed diagonally into the soil core, and the soil water was sampled by connecting the lysimeter to a 10-mL pre-evacuated sterile sampling tube (Rhizon SMS). At the sampling site in the field, the soil water was also collected with the lysimeters installed diagonally in the upper 5 cm of the soil.

Ammonium and free amino acids were separated as their 9-flourenylmethyl chloroformate derivates (Näsholm et al. 1987) by reversed-phase liquid chromatography using a Waters HPLC system (Waters 600 controller, Waters pump 60F, Waters autosampler 717), and detected with a Waters 470 fluorescence detector. The pressure was 1500 psi, and the excitation/detection wavelength was 265/330 nm. The individual components were separated on a Merck LiChroCART 250-4 Chrospher 100 RP-18 column (particle size 5 *μ*m, 40°C) using the following gradient of MeOH diluted with 1 mL tritethylamine and 1 mL HAc/1000 mL of deionized water (pH 4.2): 0–10 min 40% MeOH, 10–20 min 50% MeOH, 20–30 min 68% MeOH, and 32–35 min 100% MeOH.

### ATP and PLFA measurements

Adenosine triphosphate was extracted from the soil using the method described by Eiland (1985), but with some modifications. Soil (0.5 g fresh weight) was extracted with 20 mL of ice-cold 500 mmol/L H_2_SO_4_ and 250 mmol/L Na_2_HPO_4_ for 30 min on a rotary shaker. Following extraction, 25 *μ*L of the soil solution was transferred to 3 mL of 250 mmol/L Tris with 4 mmol/L EDTA and a pH of 7.5. Of this buffered soil solution, 50 *μ*L was transferred to a scintillation vial and 50 *μ*L of luciferase–luciferin enzyme (adenosin 5′-triphosphate [ATP] assay mix, Sigma-Aldrich, Stockholm, Sweden) added. The light output was immediately measured for 15 s in a Beckman LS6500 scintillator equipped with a single photon meter. The amount of ATP in the samples was calculated from internal standards prepared by adding 19.5 mL of ice-cold extractant and 0.5 mL of ATP standard (5.0 *μ*g per 0.5 mL, disodium salt, Sigma-Aldrich) to each soil sample prior to extraction.

Total extractable PLFAs (phospholipid fatty acids) were used as an indicator of microbial biomass, as described in Törneman et al. (2008).

### Calculations

The uptake of ^15^N was expressed as atom% excess after subtracting for atom% ^15^N in unlabeled samples. Atom% was defined as [(*R*_s_/(*R*_s_ + 1)] × 100, where R_s_ is the ratio of ^15^N to ^14^N in the sample. Atom% excess was defined as [(*R*_s_ − *R*_r_)/((*R*_s_ − *R*_r_) + 1)] × 100, where *R*_r_ is the ratio of ^15^N to ^14^N in the unlabeled sample. The uptake was calculated by multiplying the atom% excess numbers by the total N content in the root and shoot samples and in the salt residues from fumigated and unfumigated samples. The total uptake of 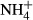, glycine, and glutamate was calculated by multiplying the fraction of ^15^N immobilized by plants and microorganisms by the total N in the 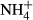, glycine, and glutamate soil pools to account for the dilution of the ^15^N label by the available N pool:


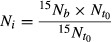


where N_i_ is the gross immobilization of 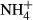 and amino acid N, N_b_ is the ^15^N taken up by the organisms and Nt_0_/^15^Nt_0_ is the ratio of total N to ^15^N in the 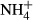, glycine, and glutamate pools within 1 h after ^15^N addition. The ^15^N addition resulted in approximately 10% increase in the 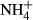 present before label application, 65% of glutamate, and 80% of glycine, respectively. Therefore, the [Nt_0_/^15^Nt_0_] factor for the amino acids was approximated to 1. No correction was made to account for the potential internal regulation of the amino acid uptake in response to the added tracer amounts (Sauheitl et al. 2009), so the calculated uptakes may overestimate uptake at ambient concentrations. Other N transformation processes than uptake of the added and available N sources within 1 h after initiation of the assay may have occurred. For instance, added ^15^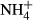 may have nitrified during the assay and ^15^N taken up as ^15^NO_3_^−^, hence, overestimating the uptake of 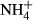 and mineralization of organic matter may have added unlabeled N to the 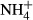 pool, hence underestimating the 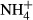 uptake. No corrections were made for those or other N transformations, but we assumed that potential enrichment and dilution of the N pools during the assay would affect N uptake in both plants and microorganisms without discrimination.

The plant uptake of ^13^C was calculated in the same way.

### Statistical analyses

The data set was tested for equality between variances (Levene′s test of equality of error variances) and for normal distribution (Kolmogorov–Smirnov and Shapiro–Wilk tests of normality). The effect of plants, the drying–rewetting treatment, and the N form on microbial N uptake was tested in three-way ANOVAs. As the N form and drying–rewetting treatment interacted, two-way ANOVAs were used to evaluate the effect of drying–rewetting and constant moisture treatments separately for the microbial N uptake. Both plant and N form had significant effects on microbial N uptake in the drying-rewetting treatment, and there was a significant interaction between the N form and the planted–unplanted treatment. A one-way ANOVA was therefore used to evaluate the plant effect on microbial N uptake for each N form separately in the drying–rewetting treatment. The effect of plants and drying–rewetting on bacterial biomass/activity (ATP) and the effect of drying–rewetting and N form on plant N uptake were tested in two-way ANOVAs. The drying–rewetting had a significant effect on the plant N uptake, and there was an interaction between the N form and the drying–rewetting treatment. Therefore, the effect of each N form on the plant N uptake was tested separately in one-way ANOVAs. The relationship between mole ^13^C excess and mole ^15^N excess in plants exposed to ^15^N-^13^C amino acids was tested in a linear regression. All statistical analyses were performed in SPSS 11.0 (SPSS Sweden, Kista, Sweden).

## Results

### Microbial ^15^N uptake and ATP-biomass/activity

**Figure 2:**
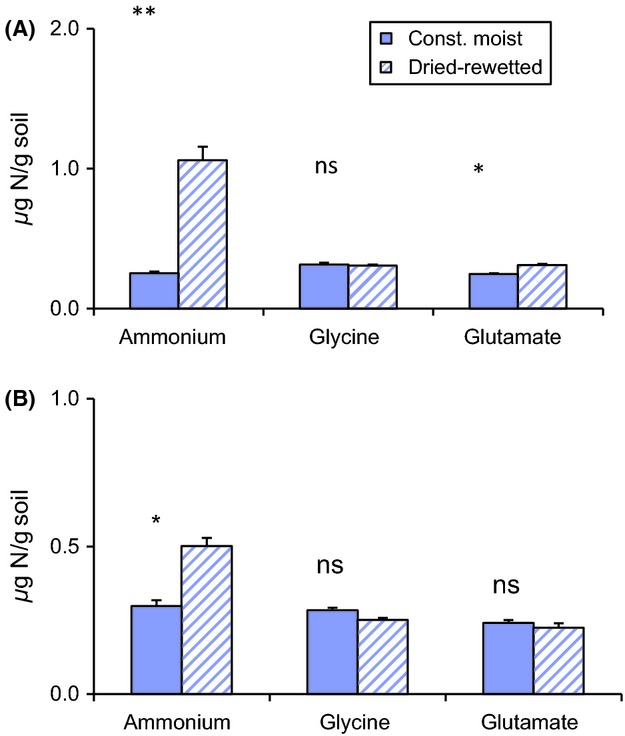
Microbial uptake of 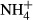, glycine, and glutamate (*μ*g N g^−1^ dw soil) in the dried–rewetted and constantly moist treatment and in the planted (A) and unplanted (B) soils. The bars represent means and standard error (*n *=* *5). One-way ANOVA; ns = *P *>* *0.05, *P <* *0.05, **P <* *0.01.

**Figure 3:**
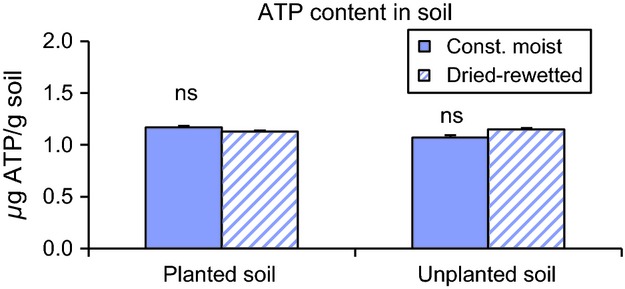
The soil ATP content (*μ*g g^−1^ dw soil) in the dried-rewetted and constant moist treatments in the planted and unplanted soils. The bars represent means and standard error (*n *=* *5). One-way ANOVA; ns = *P *>* *0.05.

Ammonium was the most important N source for the microorganisms when the moisture conditions were changed from a constant 60% of WHC to drying–rewetting (Fig. 2), especially in the planted soil, where the 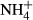 uptake was almost three times higher than the uptake of glycine and glutamate. The plant effect on microbial 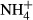 uptake was significant only in the dried and rewetted soil (*P *<* *0.01, *n *=* *5) (Fig. 2A) and not affected by changes in microbial biomass and activity, as the soil ATP content was similar in all treatments (Fig. 3). The total PLFA content was not significantly affected by the drying and rewetting treatment or by the root proliferation (106 ± 24 and 97 ± 14 nmol g^−1^ (mean ± SE) in planted and unplanted, constantly moist soil, respectively, and 102 ± 18 and 105 ± 20 in planted and unplanted, dried and rewetted soil, respectively). The microorganisms took up more 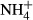 than the plants in both treatments, but the ratio of microbial to plant uptake was ten times higher in the dried–rewetted soil (70.7) than in the constantly moist soil (7.6).

The microorganisms took up the same amount of N from glycine and glutamate as from 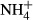 in the constantly moist soil, regardless of the presence or absence of the plant (Fig. 2). As the amount of glycine N and glutamate N taken up was independent of the drying–rewetting event (Fig. 2), microorganisms in all assays used 7–8 times more of each of the two amino acids than the plant. These observations together show no evidence for a limitation of the uptake of N by microorganisms imposed by the plant in constant or varying soil moisture conditions. Thus, one of the qualifications for competitive interactions by the plant on microorganisms was not fulfilled.

The microbial uptake of ^15^N ranged from 14 to 36% of added ^15^N, with the highest values in planted dried–rewetted soil, while the plants took up less than 2% of the added ^15^N. The total plant and microbial uptake of 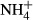 were 14% of the extractable 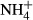 concentration in the constantly moist soil and 30% in the dried–rewetted soil (calculated from data in Figs. 1, 3 and Table 3).

**Table 3:** Soil solution (sampled with Rhizon tension lysimeters) concentrations of 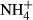, glycine, and glutamate (*μ*g × 10^−2^ N g^−1^ dw soil) in the different treatments at the start of the 24-h competition assay.

	Dried–rewetted soil			Const. moist soil		
	Ammonium	Glycine	Glutamate	Ammonium	Glycine	Glutamate
Planted soil	371 ± 11	1.12 ± 0.084	0.34 ± 0.031	226 ± 8	0.58 ± 0.029	0.19 ± 0.023
Unplanted soil	323 ± 10	1.94 ± 0.145	0.47 ± 0.058	242 ± 6	1.34 ± 0.095	0.19 ± 0.010

Values are mean ± SE (*n *=* *5).

### Plant ^15^N uptake and uptake of intact amino acids

While 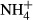 became the dominant N source for microorganisms, it became less important to plants when the soil was dried and rewetted as compared to the constantly moist soil (Figs. 1, 3). The shift was mostly an effect of the significant decrease in the 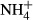 uptake (*P *<* *0.05, *n *=* *5), but there were slight increases in the glutamate uptake as well (*P *<* *0.01, *n *=* *5), in the dried–rewetted soil. The allocation of ^15^N and ^13^C to the shoots was independent of the soil moisture conditions, ranging from 33 to 40% and 24 to 43% of total uptake for ^15^N and ^13^C, respectively. The root dry biomass ranged from 25.5 mg dw to 28.9, independent of the treatment.

**Figure 4:**
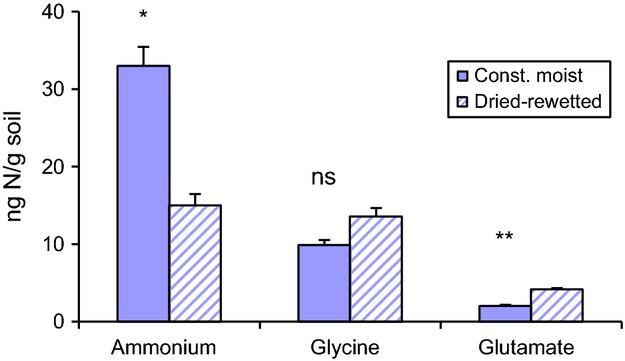
Plant uptake of 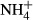, glycine, and glutamate (ng N g^−1^ dw soil) in the dried–rewetted and constantly moist treatment. The bars represent means and standard error (*n *=* *5). One-way ANOVA; ns = *P *>* *0.05, *P <* *0.05, **P <* *0.01.

The order of N source preference was 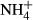, glycine, and glutamate in the constantly moist soil, and the plant took up twice as much 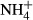 as amino acid N (Fig. 4). This uptake was one-tenth and even less (amino acids) of the uptake by the microorganisms (cf. Figs. 2, 4). As a consequence of the more than 50% reduction in the 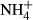 uptake as the soil was dried and rewetted, glycine and 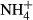 became equally used N sources. The plant reduced its acquisition of N from all three sources together by an average of 28% as the constantly moist soil was dried and rewetted (cf. Fig. 4). As the microorganisms increased their N uptake by an order of magnitude at the same change of soil conditions, the second part of the condition for resource competition, namely the limitation of the availability of N by a potential competitor, may be fulfilled.

**Figure 5:**
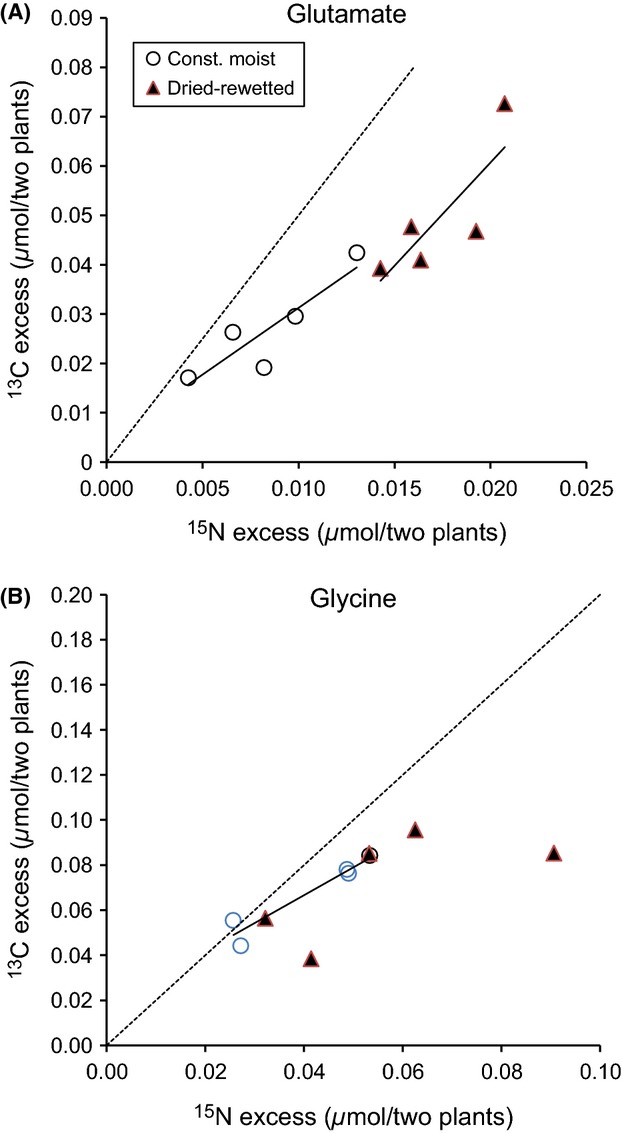
The relationship between excess ^13^C and excess ^15^N in plants from dried–rewetted and constantly moist soils treated with ^13^C_5_-^15^N-glutamate (A) and ^13^C_2_-^15^N-glycine (B). The ^13^C:^15^N ratio of added glycine (slope = 2.0) and glutamate (slope = 5.0) is shown by the broken lines, while the regressions calculated from observations of excess ^13^C versus excess ^15^N in plants are represented by unbroken lines. Glutamate-treated plants in constantly moist soil: slope = 3.23, *R*^2^ = 0.95, *P *<* *0.01, and dried–rewetted soil: slope = 4.25, *R*^2^ = 0.87, *P *<* *0.05, and glycine-treated plants in constantly moist soil: slope = 1.25, *R*^2^ = 0.89, *P *<* *0.05, and dried–rewetted soil: slope = 0.70, *R*^2^ = 0.48, *P *=* *0.19 (not shown).

Large proportions of the labeled amino acids were taken up intact, as illustrated by the linear relationship between excess ^13^C and excess ^15^N in the plant tissue (Fig. 5). A slope of 2.0 for ^13^C_2_-^15^N-glycine and 5.0 for ^13^C_5_-^15^N-glutamate would be equivalent to 100% of assimilation of ^15^N as intact amino acids. A slope of 1.25 for ^13^C versus ^15^N in glycine-treated plants in our study suggests that 62 % of the ^15^N was taken up as intact glycine or, alternatively, that there was some metabolism within the plant. Likewise, the slopes of 3.23 and 4.25 for glutamate suggest that 65 and 85% were taken up intact in the constant moist and dried–rewetted treatments, respectively (Fig. 5), or, alternatively, that there was some metabolism in the plant that varied with the soil moisture conditions. However, the mean ratio of the ^13^C-to-^15^N uptake was closer to the value of the intact tracer for glycine than for glutamate and for the constantly moist samples compared with the dried–rewetted (Table 2).

**Table 2:** The ratio of excess ^13^C-to-excess ^15^N in *F. gigantea* after the 24 h competition assay. Values are mean (*n *=* *5). The added ^13^C_2_-^15^N-glycine and ^13^C_5_-^15^N-glutamate had ^13^C:^15^N ratios of 2:1 and 5:1, respectively.

N form Treatment	^13^C_2_-^15^N-Glycine		^13^C_5_-^15^N-Glutamate	
	Const. moist	Dried–rewetted	Const. moist	Dried–rewetted
Ratio of excess ^13^C:^15^N in *F. gigantea*	1.69	1.30	3.17	2.25

### Soil amino acid and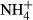 concentrations

The 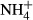 concentrations were considerably (*P *<* *0.001, *n *=* *5) higher than the concentrations of any of the amino acids in all treatments, whereas glycine and glutamate concentrations were similar (Table 3). Drying and rewetting increased the concentrations of all three N forms (*P *<* *0.001 for 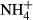 and *P *<* *0.05 for glycine and glutamate). The ratio of glycine and glutamate concentrations to the 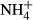 concentration was orders of magnitude larger in the field than in the experimental soil (Tables 1 and 3). In the field samples, the total concentration of the nine most abundant amino acids was similar to the 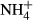 concentration (Table 1).

## Discussion

Organisms qualifying as potential competitors have to use a common and limited resource (Burkholder 1952). The first requirement was fulfilled in the experiment, as both plants and microorganisms used all three N sources. However, it was difficult to determine whether any of the sources was limiting. The 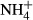 uptake in plants and microorganisms during the 24-h assay in the constant moist soil was only about 14% of the pool at the end of the assay (cf. Figs. 1, 3 and Table 3). That seems to point to 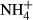 unlimited conditions, in which case, the absence of a plant effect on microbial N uptake in the constant moist soil may reflect soils without N limitation. Those conditions would be inappropriate for a rejection of the first hypothesis, which suggested competition for N in the constant moist soil by impaired microbial 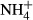 uptake in the presence of plants. However, the low degree of utilization of the 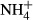 pool does not necessarily mean that the system was N unlimited, as the turnover time is not known.

Drying and rewetting of a soil is known to induce a burst of mineralization of organic N, resulting in high soil concentrations of 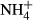 (Kieft et al. 1987; Van Gestel et al. 1993; Pulleman and Tietema 1999) and amino acids (Lipson and Monson 1998). Our experiment was no exception. Notwithstanding the fact that the soil 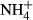 concentration increased, there was strong evidence for the drying and rewetting treatment favoring the 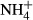 uptake in microorganisms, especially in the presence of plants. The microbial uptake increased to almost 30% (cf. Figs. 1, 3and Table 3) of the 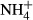 pool at the beginning of the 24-h assay, at the expense of the uptake in the plants, which decreased by 60%. The reduced plant uptake of 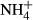 in the dried and rewetted soil may depend on superior uptake by fast-growing bacteria and microfungi, which have higher surface-to-volume ratios and growth rates than plants, triggered by rhizodeposition and exudates from the plants (Henriksen and Breland 1999; Neuman and Römheld 2001; Kaiser et al. 2011). Those changes in the 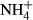 uptake pattern in combination with the simultaneous shift toward an amino acid dominated N uptake in the plants, supposedly to partly compensate for the negative effects of the increased microbial 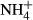 immobilization, may be taken as a pretext for a temporary competition for 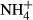 under conditions of net mineralization exceeding immobilization.

The microbial 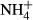 uptake was stimulated by the drying–rewetting treatment also in the absence of roots, suggesting that concentrations of easily degradable substrate with high C:N ratios increased when the soil was dried and then moistened, possibly by release of physically protected organic matter (Van Gestel et al. 1993; Pulleman and Tietema 1999) and extraction of C when water was added. This result is consistent with previous studies that also found increased microbial ^15^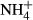 immobilization from 24 h (Pulleman and Tietema 1999; Bengtsson et al. 2003) to 1 week (Schimel et al. 1989) after rewetting a dry soil. The lack of correspondence between variations in microbial N uptake and the ATP content in soil may depend on the insensitiveness of the ATP measurement to subtle variations in microbial activity (Contin et al. 2000). In a study by Raubuch et al. (2002), soil drying and rewetting was found to lower the ATP content in the soil but increase the respiration rates compared with a constantly moist soil, suggesting a decrease in microbial biomass rather than a lowered activity. Compared with our experiment, their soils were dried fast (15 h) and at high temperature (40°C), which may have caused cell lysis and consequently a greater reduction in microbial biomass and ATP content.

Amino acid N is potentially a more cost-effective N source under conditions when the leaf net CO_2_ uptake decreases because of stomatal closure, for example during drought (Cornic and Massacci 1996; Schmidt and Stewart 1999). Drought causes a decline in the activity of some enzymes, for example nitrate reductases (Cornic and Massacci 1996), and induces others, such as root proteases, facilitating amino acid uptake (Kohli et al. 2012). That may provide an additional explanation to the higher amino acid uptake in the dried–rewetted soil. The plant seemed to have a preference for glycine-to-glutamate, which is in agreement with previous studies on preferences for less complex amino acids (Lipson et al. 1999; Weigelt et al. 2005; Harrison et al. 2007), whereas the microorganisms showed no discrimination between the two. This suggests that *F. gigantea* used the different amino acids independent of the immobilization by the microorganisms. The short-term uptake of glycine-^15^N was similar in *F. gigantea* and grass species from other ecosystems (Henry and Jefferies 2003; Näsholm et al. 2009), but the drying–rewetting effect on the amino acid uptake was opposite to that found in the alpine sedge *Kobresia myosuroides* (Lipson and Monson 1998). That may have been the result of a longer drying period (45 days) for the soil with *K. myosuroides*, causing more damage to the roots than in our study.

The lower ^13^C:^15^N ratio in *F. gigantea* in the dried and rewetted treatments compared with the constant moist soil can be explained by either an uptake of mineralized amino acid-^15^N parallel to the intact amino acids, or by a higher amino acid catabolism and respiration by plants in the dried–rewetted treatment. Under conditions when carbohydrates are in low supply, for example when water is limiting, plants can deaminate glutamate and catabolize glycine and use the C as an energy source (Buchanan et al. 2000). The ^15^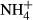, produced from the deamination, may be used to synthesize new amino acids, while some of the ^13^C may be lost by respiration, thus reducing the ^13^C:^15^N ratio in the plant. The slopes of the regression lines for excess ^13^C versus excess ^15^N in the plants were not as steep as the slope of the intact tracer, which suggests that the proportion of ^13^C-to-^15^N decreased with the plant uptake of ^15^N. This would mean that the proportion of mineralized amino acid-^15^N in relation to ^15^N from intact amino acids taken up by the plants increased with increasing ^15^N uptake or, alternatively, that the amino acid catabolism increased with increasing ^15^N uptake. If 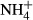, for various reasons, is more easily taken up by plants than amino acids (Nordin et al. 2001), then, the plant uptake of mineralized to intact amino acid ^15^N would be positively related to the ^15^N uptake in plants. On the other hand, it is well documented that increased plant nutrient uptake rates also increase the energy demand (Marschner 2002). That would provide an alternative explanation to the lower excess ^13^C:^15^N ratios in plants with higher ^15^N uptake, as amino acid-^13^C may be used as energy source.

Most short-term studies show that microorganisms take up a larger part of 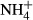 than plants at constant soil moisture conditions (Jackson et al. 1989; Schimel et al. 1989; Zak et al. 1990; Bardgett et al. 2003), but plants may sometimes acquire more 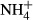 than microorganisms depending on, for example, root length (Xu et al. 2011) and C/N ratio of the soil (Månsson et al. 2009). *F. gigantea* took up twice as much 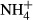 as microorganisms did in another competition experiment with soil from the same oak-dominated site and under the same experimental conditions as here (Månsson et al. 2009), but with one observed difference: the average 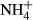 concentration at the start of that assay was five times higher (8.13 *μ*g g^−1^) than in the present study. Microorganisms suffered from competition with the plant for 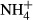 in that assay. It is possible that the ratio of 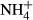 uptake between plant and microorganism varies with the 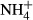 concentration and that microorganisms take up a larger proportion in 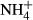 limited soils, as depicted by Schimel and Bennett (2004) and observed by, for example Bardgett et al. (2003).

At the oak-dominated site, the *in situ* gross 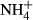 immobilization rate was autocorrelated within a range of 2.7 m and varied spatially by two orders of magnitude within a 100 m^2^ plot (Bengtson et al. 2006), suggesting that the small-scale spatial pattern of competitive outcome may be quite heterogeneous. As the half-lives of 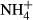 and common amino acids is less than 24 h even at soil temperatures below 10°C (Henry and Jefferies 2003), the spatial pattern may also have a profound short-term temporal variation. Temporal changes of soil conditions add another dimension of heterogeneity in competition. For instance, short pulses of carbon and nutrients following addition of labile carbon and periods of drying and wetting tend to favor N uptake in microorganisms at the expense of the plant (Dunn et al. 2006; Månsson et al. 2009; this study). This immobilization is probably temporal, as turnover time is short for microorganisms (few days; Schmidt et al. 2007) and dissolved organic carbon, the main source of respired carbon (few hours; Bengtson and Bengtsson 2007). Portions of the mobilized N are suggested to be continuously relocated to the plant in a mainly unidirectional flow and immobilized in roots and aboveground tissues over longer periods (Kuzyakov and Xu 2013).

In conclusion, a test of competition between a plant and microorganisms for 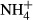 and amino acids was performed, in which not only data for N uptake is considered but also conditions for an equal soil environment for microorganisms in the presence and absence of the plant. In addition, for the first time competition between a plant and microorganisms for N was tested by fulfilling a necessary condition for resource competition, namely the removal of one of the potential competitors to observe the detriment of its presence to the other. This new competition assay gave no evidence for competition for 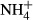, glycine, or glutamate when the soil moisture was kept constant at 60% WHC. But the assay showed that microorganisms increase their 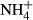 uptake in the presence of plants and plants decrease theirs when a drier soil becomes rewetted. Some of the reduction was compensated by an increased uptake of glycine and glutamate N, but plants were still taking up less than a tenth of the uptake by microorganisms. Far more 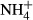 was taken up by microorganisms in the presence of plants than expected from its increase in soil solution concentration following rewetting, suggesting some triggering function associated with the roots. The flexibility in plant and microbial uptake of N in response to short-term soil moisture fluctuations demonstrates the importance of including transient soil conditions in experiments on resource competition between plants and soil microorganisms.
